# Supplementary material for: Fragility Fractures and Imminent Fracture Risk in the Spanish Population: A Retrospective Observational Cohort Study
Source: J Clin Med. 2021 Mar 5;10(5):1082. doi: 10.3390/jcm10051082 (PMC7961522; doi:10.3390/jcm10051082)
Supplement: Supplementary file 1 [file jcm-10-01082-s001.pdf]

Journal of
Clinical Medicine

MDPI

TITLE: ANALYSIS OF IMMINENT FRACTURE RISK IN THE SPANISH POPULATION: A RETROSPECTIVE OBSERVATIONAL COHORT STUDY.

SUPPLEMENTARY

Supplementary Table S1. Fracture codes according to ICD-9.. 2

Supplementary Table S2. Incidence rate of subsequent fracture events by type of index fracture and follow-up period (years). 3

Supplementary Table S3. Relative Risk of subsequent fracture events by age, sex and index fracture site. 4

Journal of
Clinical Medicine

MDPI

Table S1. Fracture codes according to ICD-9.

**Fracture**	**ICD-9 code**
Hip fracture	820.0, 820.2, 733.14
Spine fracture	805.0, 805.2, 805.4, 805.8, 806.0, 806.2, 806.4, 806.8, 733.13
Pelvic fracture:	808.0, 808.2, 808.4, 808.8
Femur (thigh) fracture	821.0, 821.2, 733.15
Humerus (arm) fracture:	812.0, 812.2, 812.4, 733.1
Wrist fracture	813.4, 733.12

ICD-9 9th revision of the International Statistical Classification of Diseases and Related Health Problems

Journal of
Clinical Medicine

Table S2. Incidence rate of subsequent fracture events by type of index fracture and follow-up period (years).

	N (at risk)	person-years	SsFxs	IR x 10000 PY	95% CI
**Hip**					
All follow-up	431	909.6	37	406.8	(294.7 - 561.4)
≤1 year since 1st Fx	431	390.6	16	409.7	(251.0 - 668.7)
1-2years since 1st Fx	368	354.3	12	338.7	(192.4 - 596.5)
>2years since 1st Fx	340	164.8	9	546.0	(284.1 - 1049.4)
**Pelvis**					
All follow-up	74	164.4	7	425.8	(203.0 - 893.1)
≤1 year since 1st Fx	74	70.6	3	424.7	(137.0 - 1316.8)
1-2years since 1st Fx	67	64.6	3	464.1	(149.7 - 1439.0)
>2years since 1st Fx	62	29.1	1	343.2	(48.4 - 2436.5)
**Vertebral**					
All follow-up	94	219.5	8	364.5	(182.3 - 728.9)
≤1 year since 1st Fx	94	92.1	0	0.0	.
1-2years since 1st Fx	92	87.1	6	688.6	(309.4 - 1532.8)
>2years since 1st Fx	84	40.3	2	496.7	(124.2 - 1986.2)
**Proximal humerus**					
All follow-up	264	620.9	19	306.0	(195.2 - 479.8)
≤1 year since 1st Fx	264	257.4	5	194.3	(80.9 - 466.8)
1-2years since 1st Fx	253	246.6	11	446.1	(247.1 - 805.6)
>2years since 1st Fx	236	116.9	3	256.5	(82.7 - 795.4)
**Wrist**					
All follow-up	506	1196.7	28	234.0	(161.6 - 338.9)
≤1 year since 1st Fx	506	494.6	15	303.3	(182.8 - 503.0)
1-2years since 1st Fx	483	471.7	10	212.0	(114.1 - 394.1)
>2years since 1st Fx	461	230.4	3	130.2	(42.0 - 403.6)

Fx: index fracture. SsFx: subsequent fracture event. IR: incidence rate. PY: person year. CI: confidence interval.

Journal of
Clinical Medicine

MDPI

Table S3. Relative Risk of subsequent fracture events among those with previous fracture compared with the general population by age, sex and index fracture site.

	RR (95%CI)*	p-value
All individuals	1.80 (1.46-2.21)	<0.01
Male	2.49 (1.39-4.45)	<0.01
Female	1.73 (1.39-2.16)	<0.01
50-59years	2.67 (1.19-6.02)	0.02
60-69years	1.56 (0.69-3.50)	0.29
70-79years	2.56 (1.70-3.84)	<0.01
80+years	1.58 (1.21-2.05)	<0.01
Hip	1.62 (1.15-2.27)	<0.01
Pelvis	3.34 (1.83-6.08)	<0.01
Vertebral	2.64 (1.36-5.14)	<0.01
Prox. humerus	1.26 (0.68-2.31)	0.47
Wrist	1.83 (1.25-2.70)	<0.01

**Adjusted RateRatio estimated using Poisson regression models that include age and sex as covariates*
